# Supplementary material for: Region-specific denoising identifies spatial co-expression patterns and intra-tissue heterogeneity in spatially resolved transcriptomics data
Source: Nat Commun. 2022 Nov 14;13:6912. doi: 10.1038/s41467-022-34567-0 (PMC9663444; doi:10.1038/s41467-022-34567-0)
Supplement: Supplementary file 1 — Supplementary Information [file 41467_2022_34567_MOESM1_ESM.pdf]

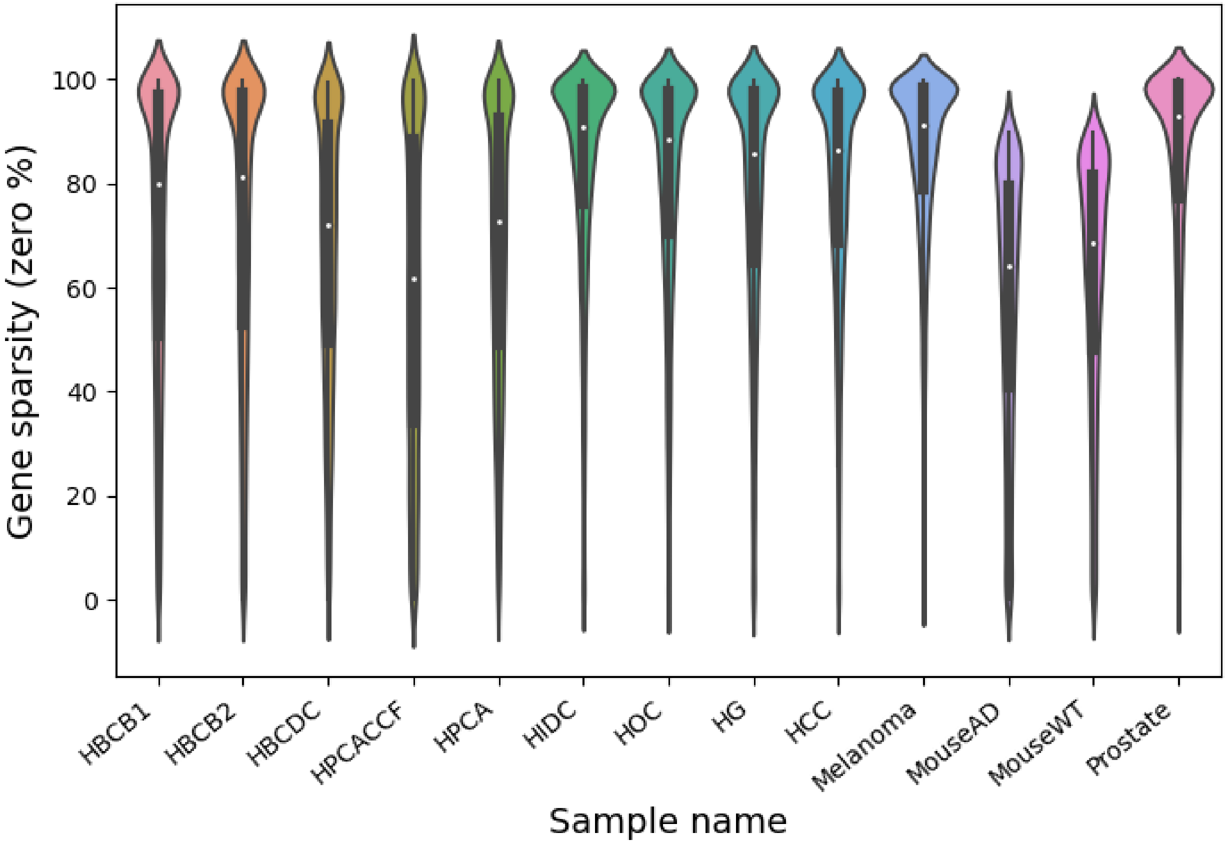

Supplementary Figure 1. Violin plots showing ST's gene-level sparsity. (n=33473 spots over 13 independent samples, Supp. Table 1). X-axis indicates sample name. Y-axis shows the distribution of the percentage of spots having zero values for each gene. Each violin plot includes a box plot. Boxplots are defined with center line (median), box limits (upper and lower quartiles) and whiskers that extend at most 1.5 times of the interquartile range.

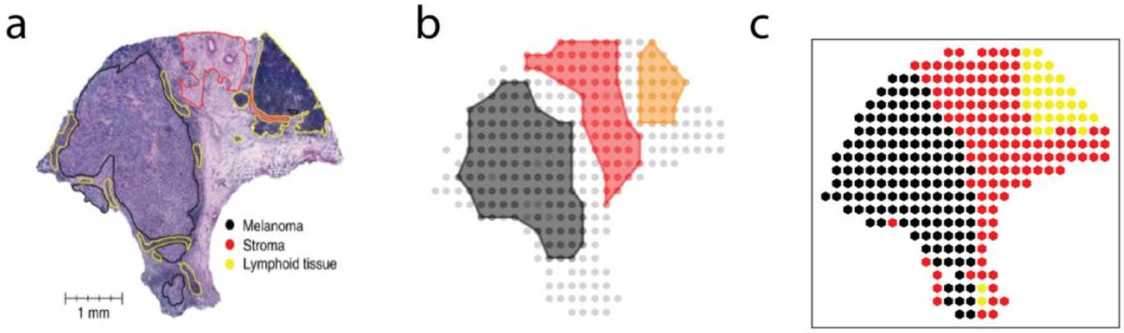

Supplementary Figure 2. Comparing melanoma's region assignments using MIST and BayesSpace. a manual annotation from human expert. **b** Spots assigned to regions by MIST. **c** Clusters assigned by BayesSpace.

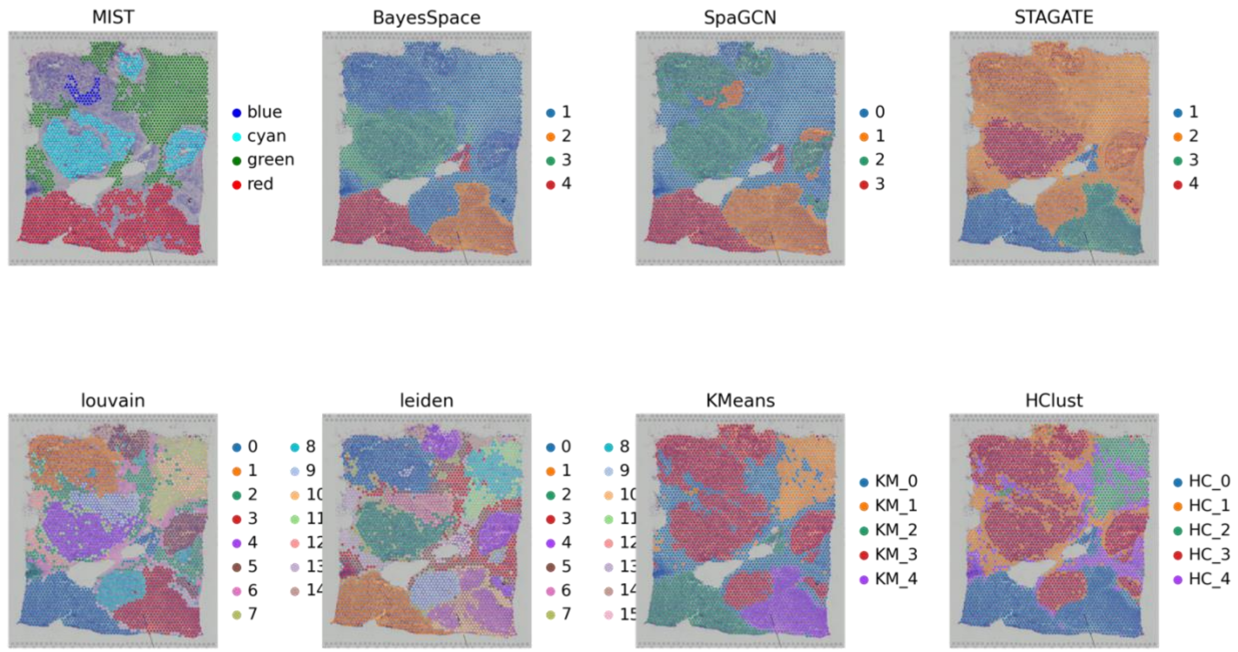

Supplementary Figure 3. Comparing spots assigned to regions by MIST and other methods on a human breast cancer sample (HBCB1, supp. Table1) with H&E staining as the background. Each color represents a different cluster, and color schemes are not matched across models.

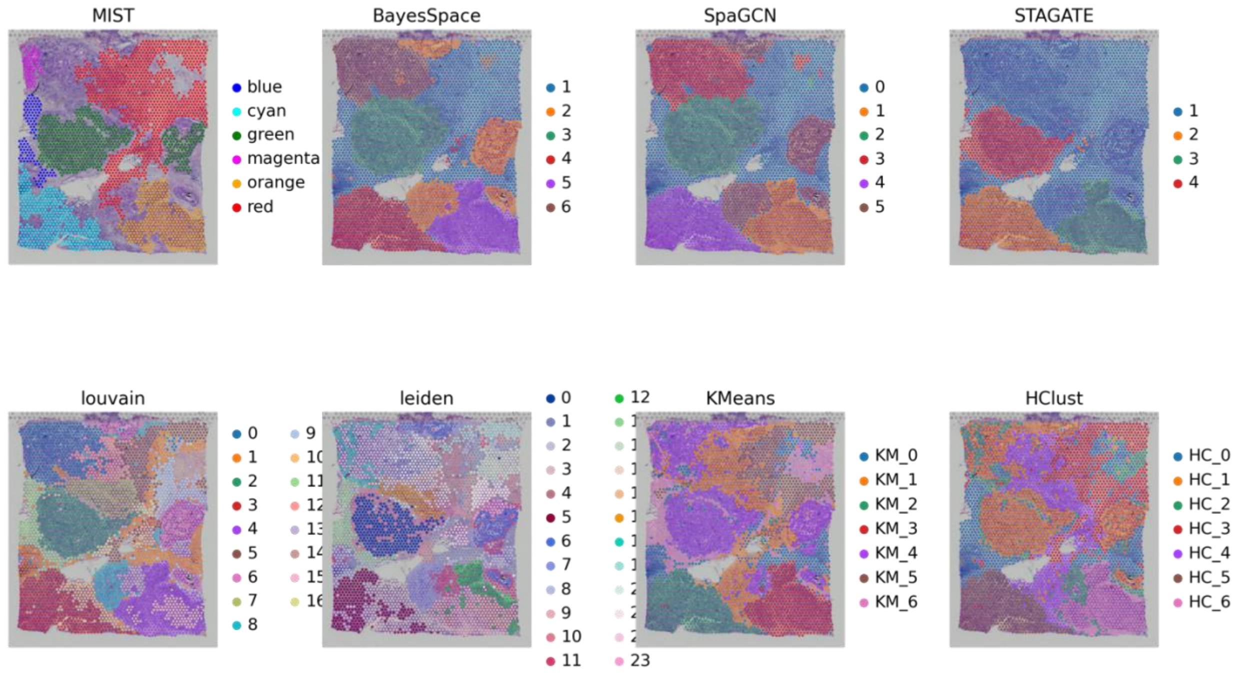

Supplementary Figure 4. Comparing spots assigned to regions by MIST and other methods on a human breast cancer sample (HBCB2, supp. Table1) with H&E staining as the background. Each color represents a different cluster, and color schemes are not matched across models.

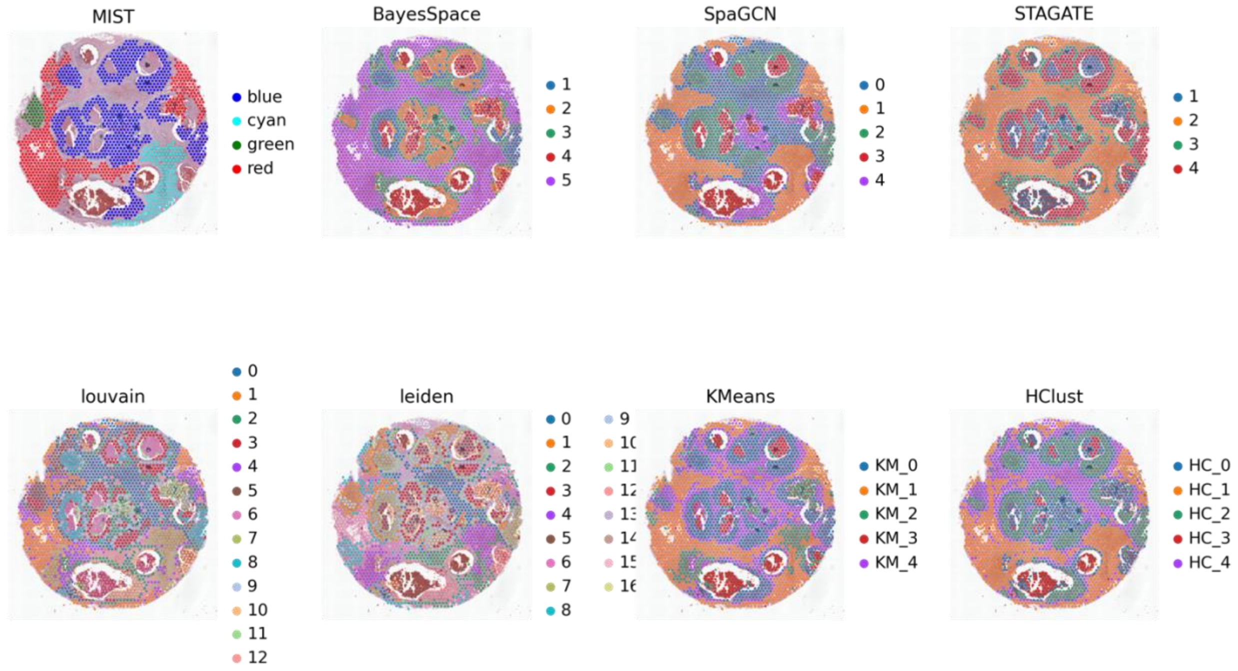

Supplementary Figure 5. Comparing spots assigned to regions by MIST and other methods on a human breast cancer ductal carcinoma FFPE sample (HBCDC, supp. Table1) with H&E staining as the background. Each color represents a different cluster, and color schemes are not matched across models.

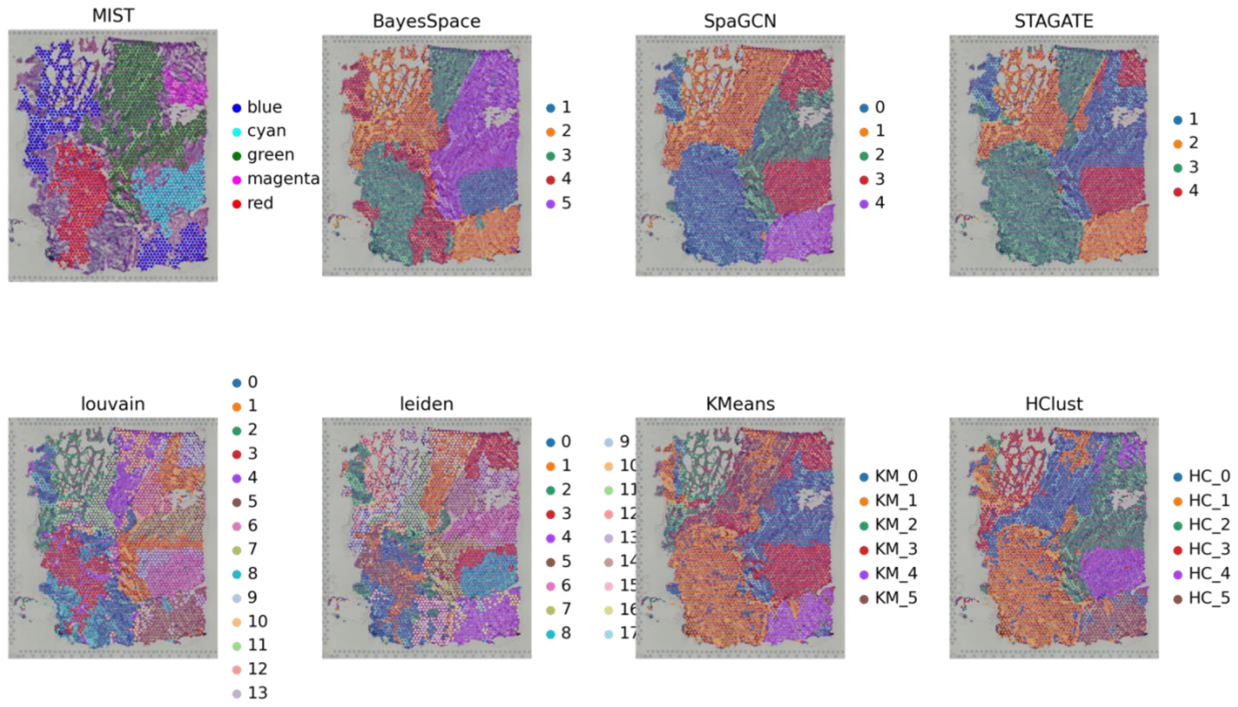

Supplementary Figure 6. Comparing spots assigned to regions by MIST and other methods on a human glioblastoma sample (HG, supp. Table1) with H&E staining as the background. Each color represents a different cluster, and color schemes are not matched across models.

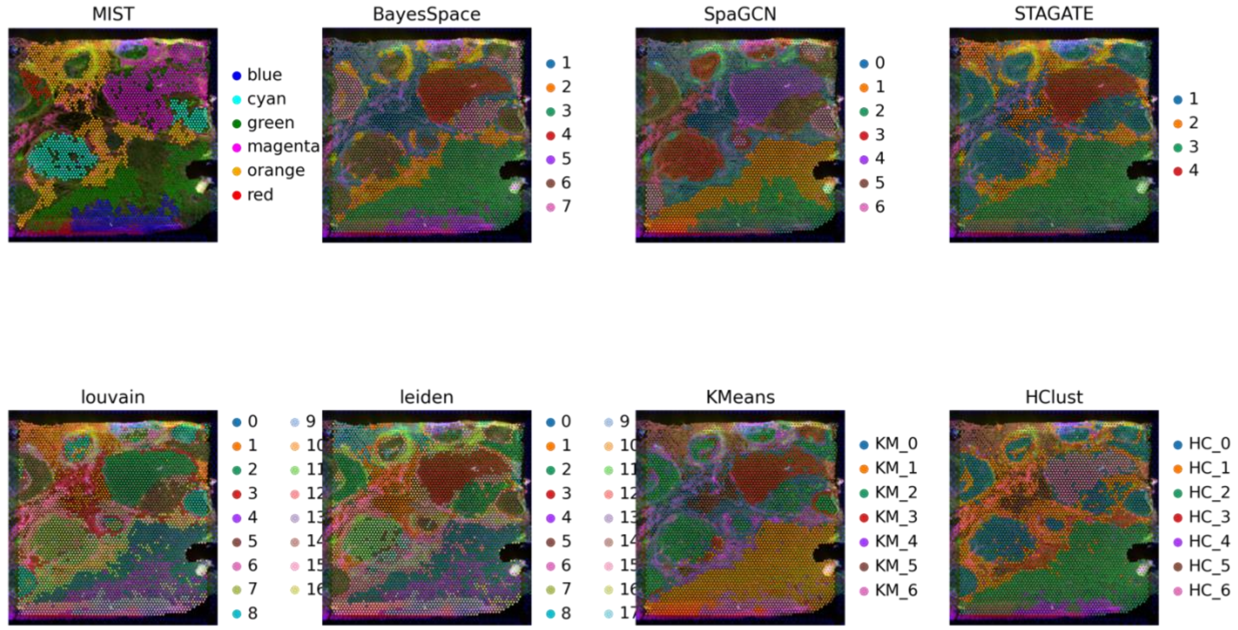

Supplementary Figure 7. Comparing spots assigned to regions by MIST and other methods on a human invasive ductal carcinoma sample (HIDC, supp. Table1) with H&E staining as the background. Each color represents a different cluster, and color schemes are not matched across models.

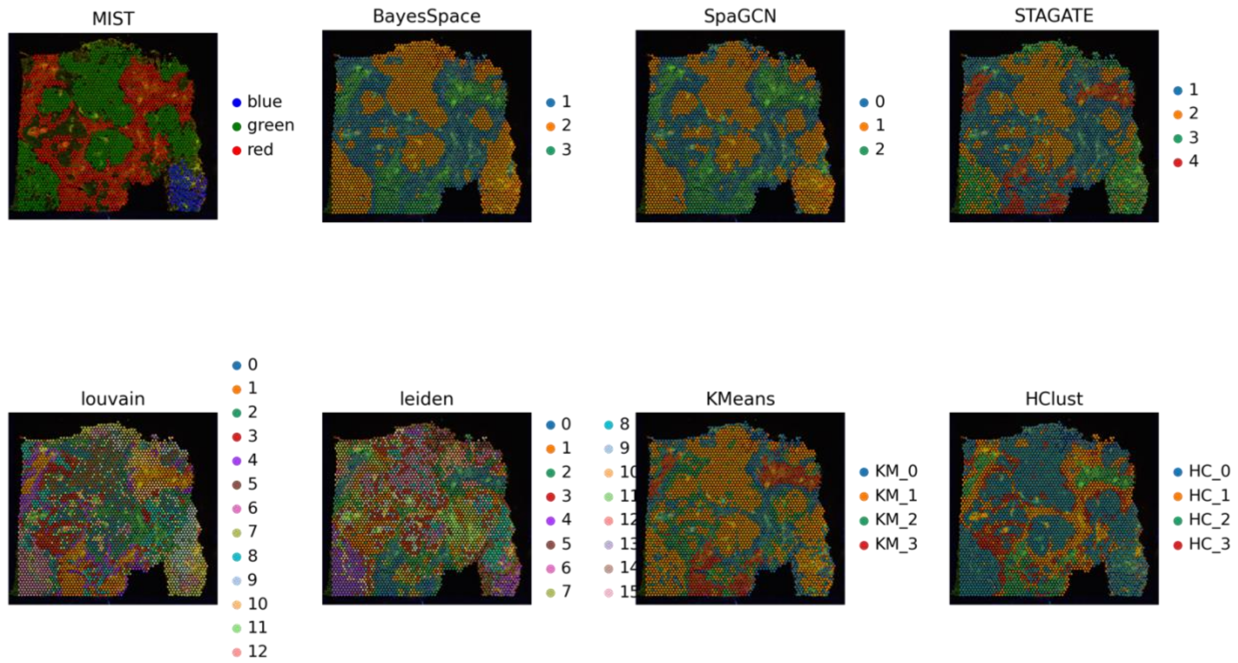

Supplementary Figure 8. Comparing spots assigned to regions by MIST and other methods on a human ovarian cancer sample (HOC, supp. Table1) with H&E staining as the background. Each color represents a different cluster, and color schemes are not matched across models.

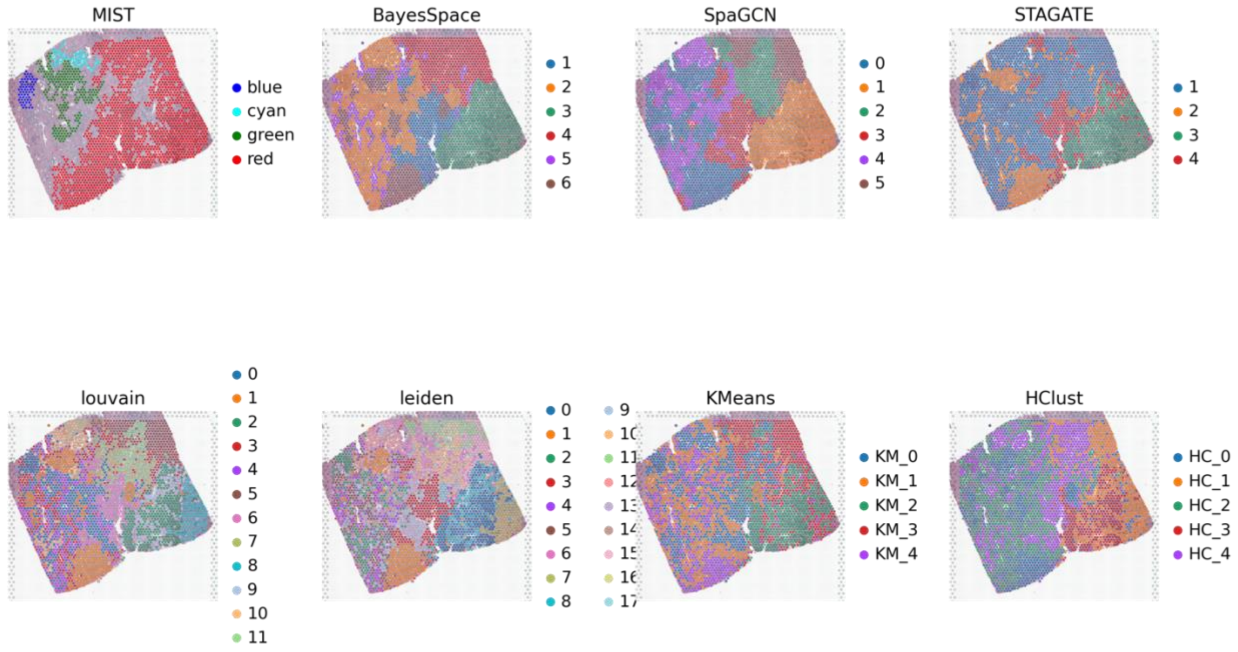

Supplementary Figure 9. Comparing spots assigned to regions by MIST and other methods on a human prostate cancer acinar cell carcinoma FFPE sample (HPCACCF, supp. Table1) with H&E staining as the background. Each color represents a different cluster, and color schemes are not matched across models.

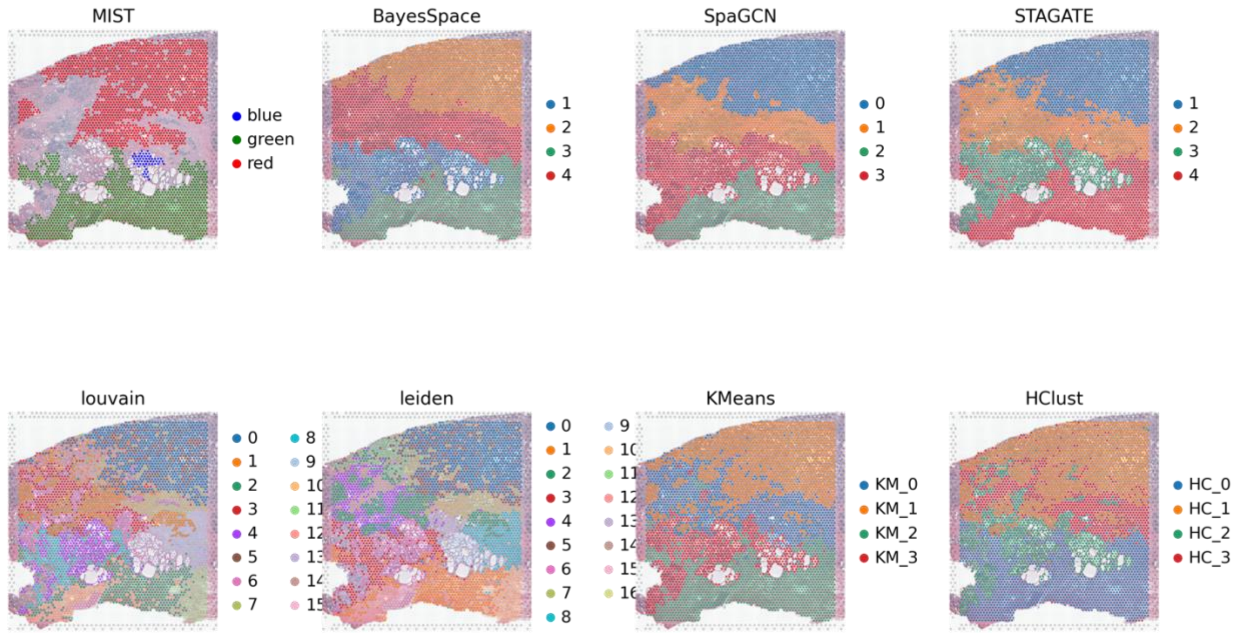

Supplementary Figure 10. Comparing spots assigned to regions by MIST and other methods on a human prostate cancer adenocarcinoma FFPE sample (HPCA, supp. Table1) with H&E staining as the background. Each color represents a different cluster, and color schemes are not matched across models.

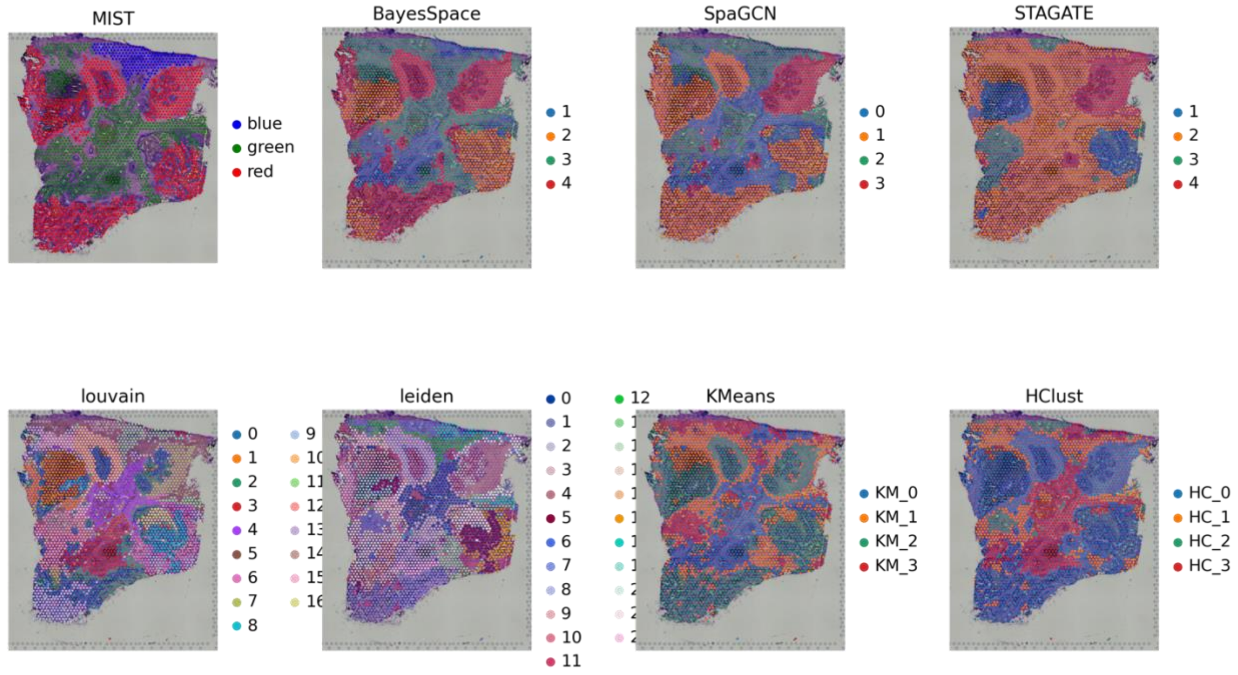

Supplementary Figure 11. Comparing spots assigned to regions by MIST and other methods on a human colorectal cancer (HCA, supp. Table1) with H&E staining as the background. Each color represents a different cluster, and color schemes are not matched across models.

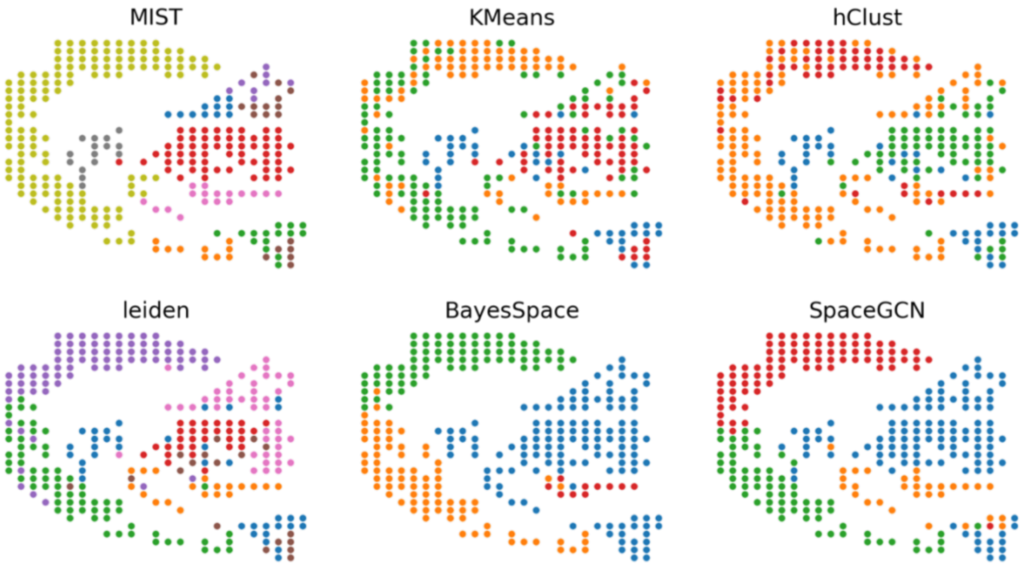

Supplementary Figure 12. Comparing spots assigned to regions by MIST and other methods on a mouse brain sample with Alzheimer's disease (MouseAD, supp. Table1). Each color represents a different cluster, and color schemes are not matched across models.

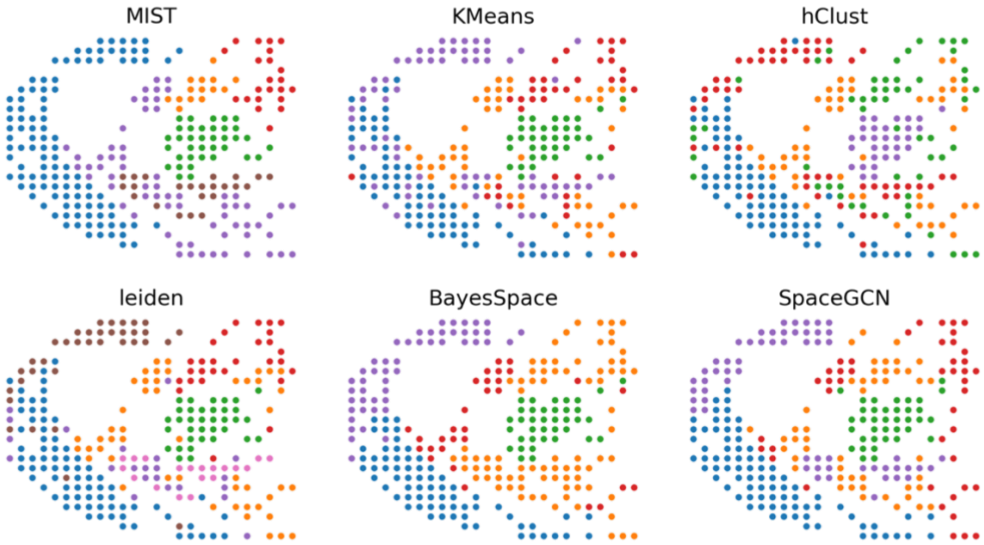

Supplementary Figure 13. Comparing spots assigned to regions by MIST and other methods on a mouse wildtype brain sample (MouseWT, supp. Table1). Each color represents a different cluster, and color schemes are not matched across models.

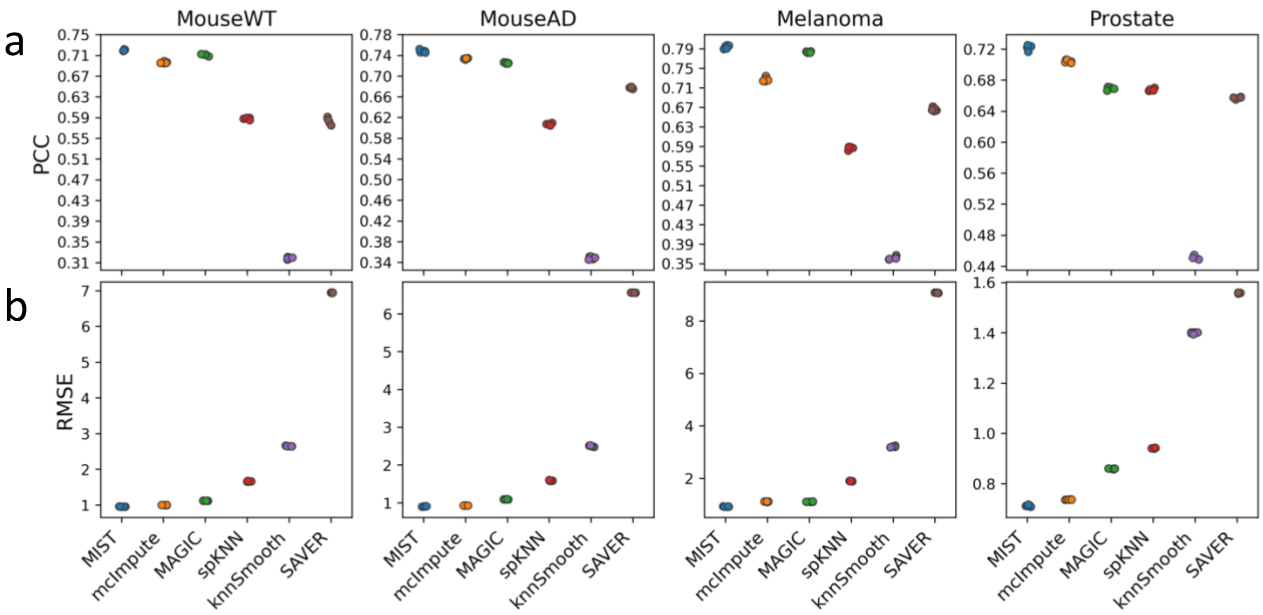

Supplementary Figure 14. Holdout test performance with SAVER on four datasets. a-b Holdout experiment performance across multiple data sets using metrics (a) Pearson's correlation coefficient, PCC and (b) Rooted Mean Square Error, RMSE. Each column is an individual data set. Points in the same color indicate the 5 non-overlap fold of tests for each model.

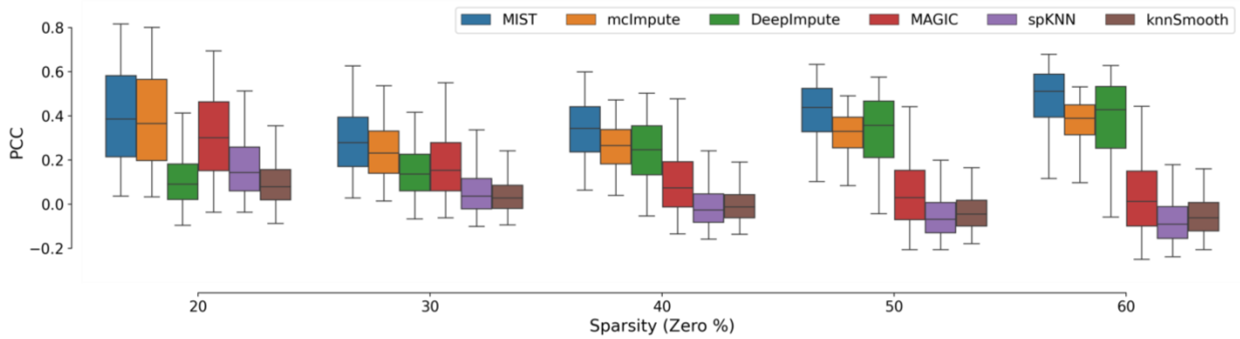

Supplementary Figure 15. Boxplot of gene-level performance of each model represented by the Pearson's correlation coefficient (PCC, y-axis). X-axis groups genes by sparsity level (zero-value percentage) of 20% (n=2078), 30% (n=4061), 40% (n=5761), 50% (n=8077) and 60% (n=6078). Center line indicates the median values. Box limits are lower and upper quartiles. Whiskers extends to at most 1.5x interquartile of PCC values with outliers removed.

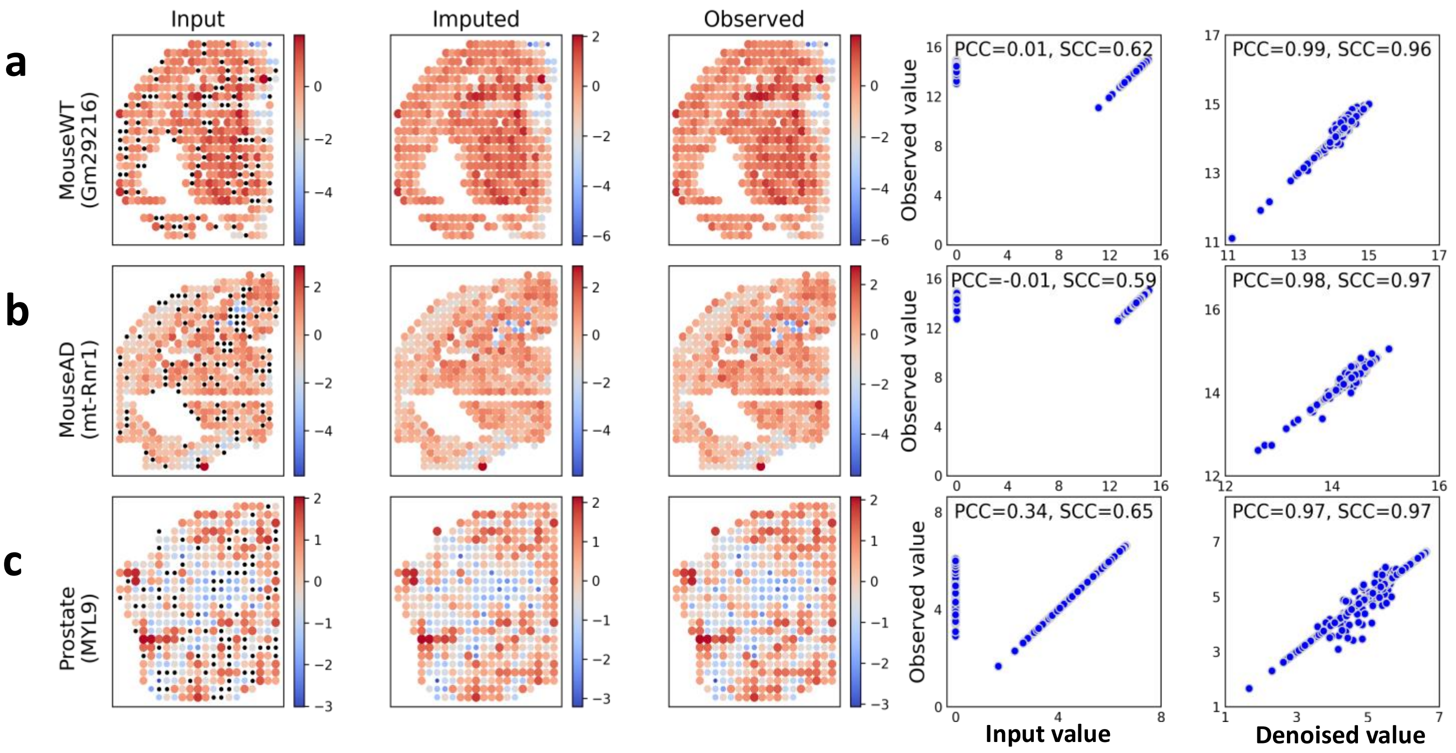

Supplementary Figure 16. MIST recovered gene expression patterns in holdout experiments. **a** Expression patterns recovered for gene Gm29216 in the Mouse WT brain. **b** Expression patterns recovered for gene mt-Rnr1 in the Mouse AD brain. **c** Expression patterns recovered for gene Gm29216 in the Prostate sample. Column 1 is the spatial pattern of the input to MIST with random non-zero values held out. Column 2 is the denoised gene expression pattern. Column 3 is the original observed gene expression pattern. Column 4 shows the correlation between input values and observed values. Column 5 shows the correlation between the denoised expression values and the observed expression values.

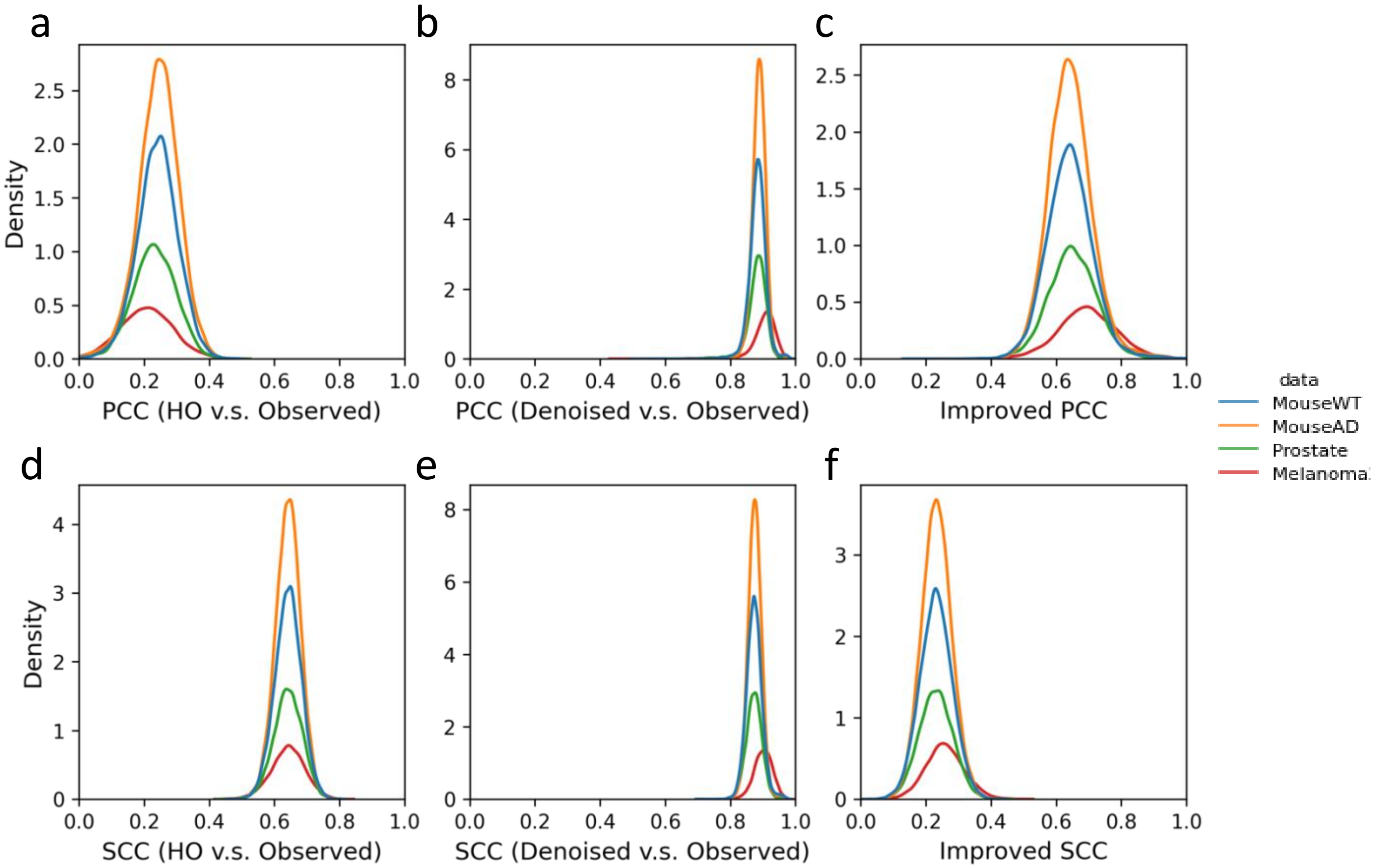

Supplementary Figure 17. Density distribution of improved Pearson's correlation coefficient (PCC) and Spearman's correlation coefficient (SCC) in holdout experiments. a PCC distribution of HO vs. Observed non-zero gene expression. **b** PCC distribution of denoised vs. Observed non-zero gene expression. **c** Distribution of improved PCC for nonzero values of each held out gene. **d** SCC distribution of HO vs. Observed non-zero gene expression. **e** SCC distribution of denoised vs. Observed non-zero gene expression. **f** Distribution of improved SCC for nonzero values of each held out gene.

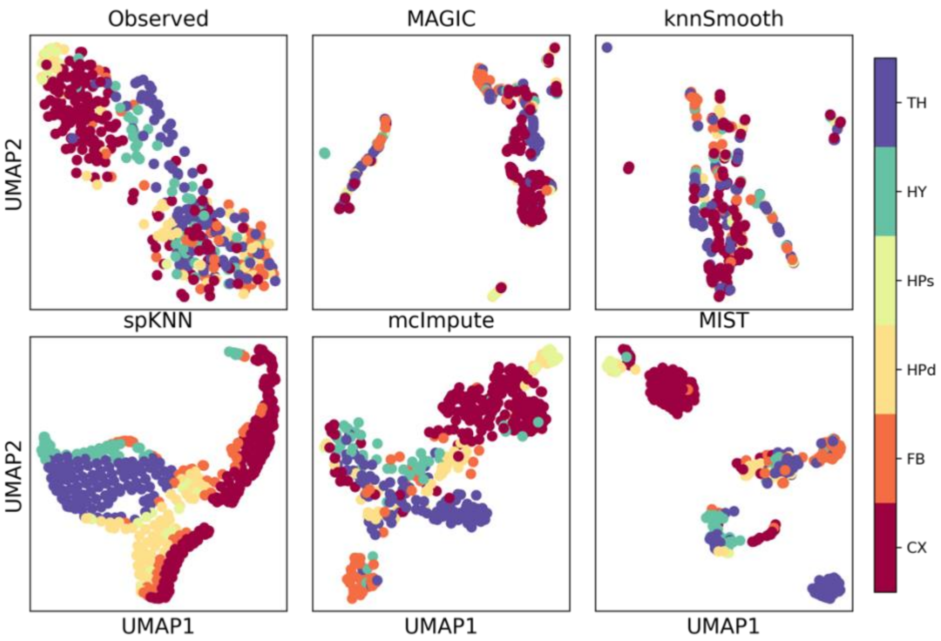

Supplementary Figure 18. UMAP visualization of Mouse WT brain using data denoised by different algorithms. Each compared algorithm or the original observed data is labeled on the top of each panel.

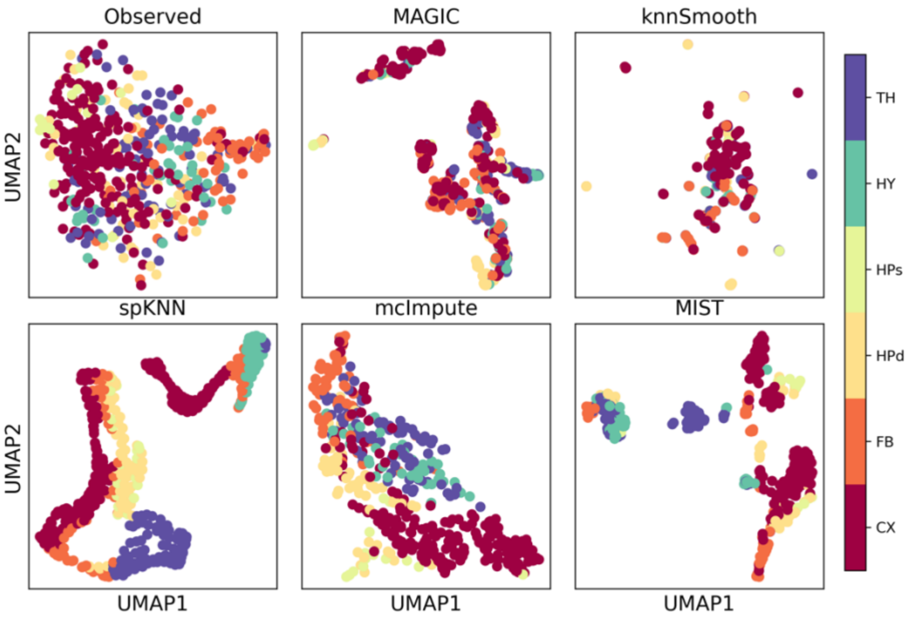

Supplementary Figure 19. UMAP visualization of Mouse AD brain using data denoised by different algorithms. Each compared algorithm or the original observed data is labeled on the top of each panel.

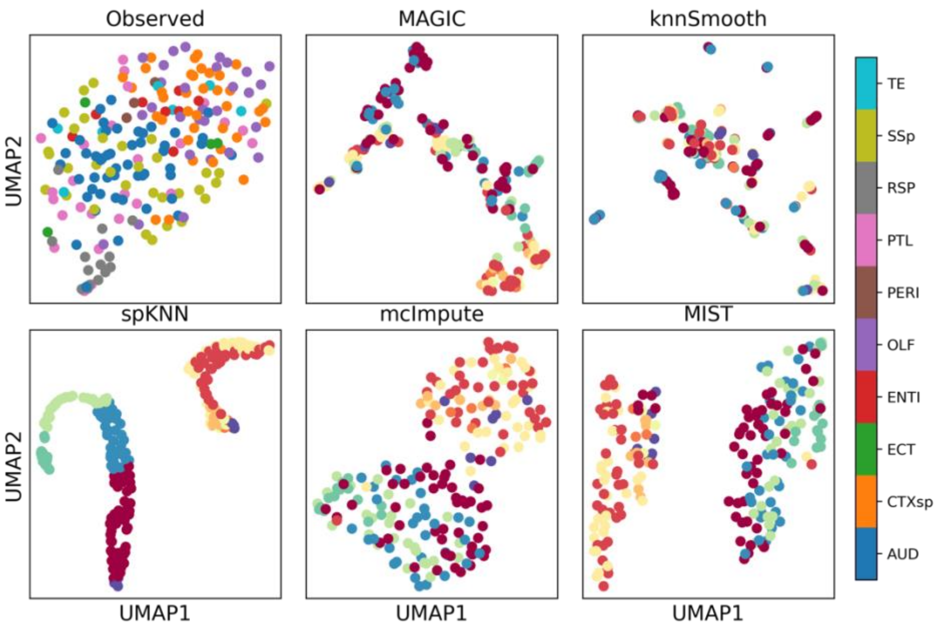

Supplementary Figure 20. UMAP visualization of Mouse AD Cortex using data denoised by different algorithms. Each compared algorithm or the original observed data is labeled on the top of each panel.

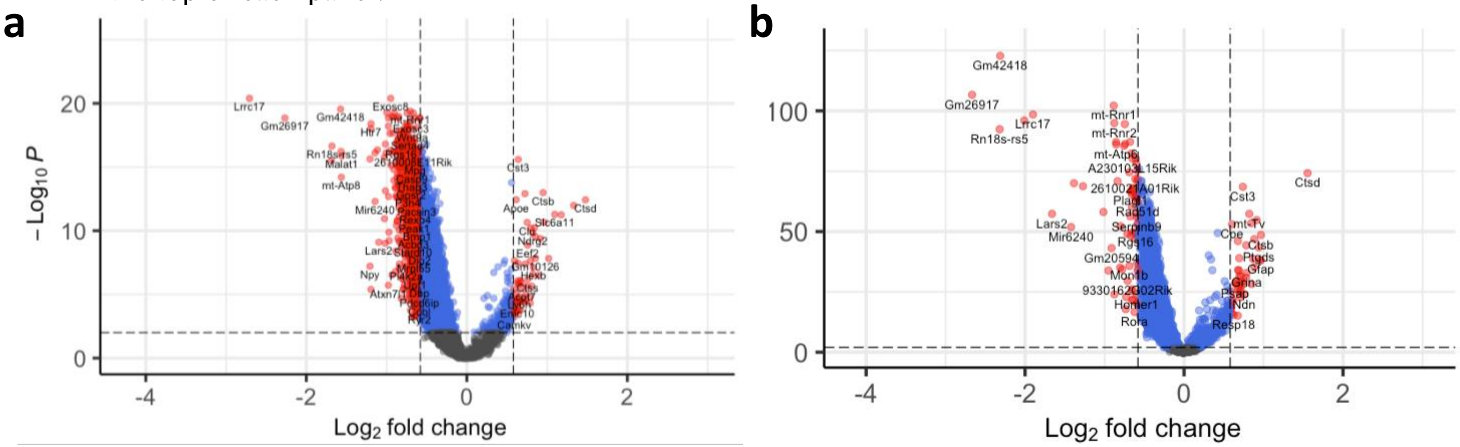

Supplementary Figure 21. Volcano plots of AD vs. WT differential expression analysis. a Volcano plot of differential gene expression analysis results comparing AD vs. WT mouse brain using Cluster 1 MIST-denoised data. **b** Volcano plot of differential gene expression analysis results comparing AD vs. WT mouse brain using Cluster 2 samples. Red dots in both panels are significant genes with absolute log2 fold change > 0.58 and FDR < 0.01 (two-sided Wilcoxon rank-sum test and adjusted for multiple comparisons). Blue dots are insignificant genes that didn't pass either fold-change threshold or FDR threshold.

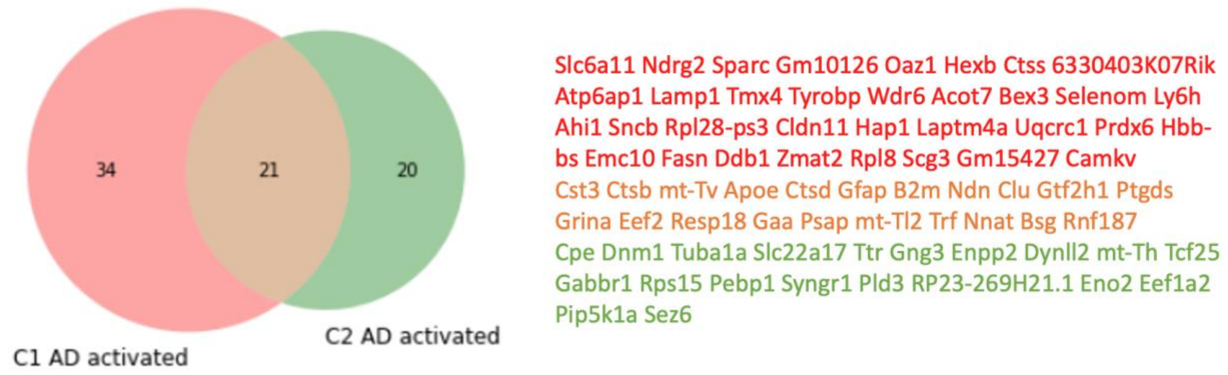

Supplementary Figure 22. Consensus and difference of AD activated genes between spatial cortex clusters. Circle and gene symbols in red color indicate genes that are only activated in Cluster 1 (C1, CTXsp, OLF, ENTI, TE, ECT and PERI). Circle and gene symbols in green are genes that are only activated in Cluster 2 (C2, AUD, PTL, RSP, SSp). Orange color indicates AD activated genes that are shared across these two spatial cortex clusters. Source data are provided with the paper in the Source Data file.

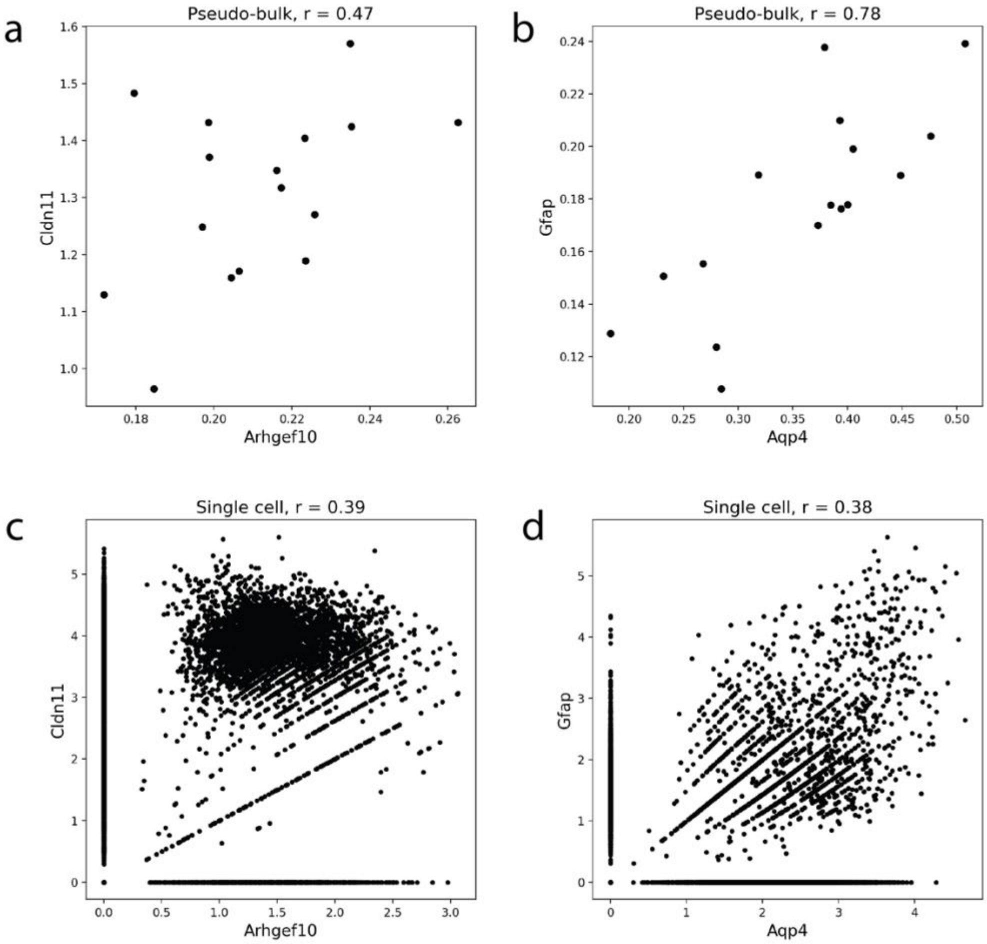

Supplementary Figure 23. Co-expression patterns of Cldn11-Arhgef10 and Gfap-Aqp4 in an additional single-cell mouse brain cohort. a-b pseudo-bulk-level co-expression patterns of Cldn11-Arhgef10 (a) and Gfap-Aqp4 (b). **c-d** single-cell-level co-expression patterns of Cldn11-Arhgef10 (c) and Gfap-Aqp4 (d). Correlation score is derived from Spearman's rank correlation test. Source data for pseudo-bulk expression values are provided with the paper in the Source Data file.

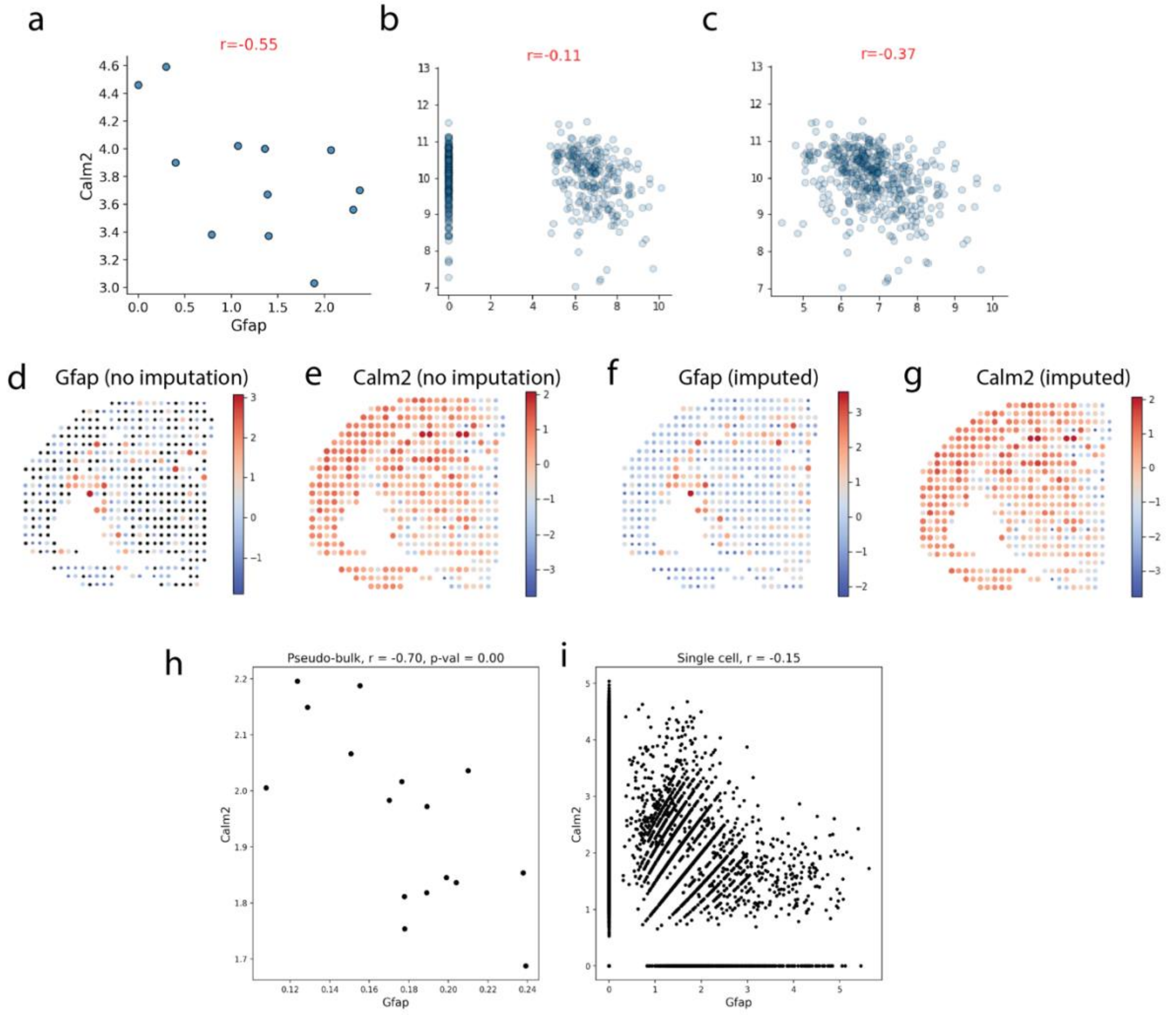

Supplementary Figure 24. Negative correlation patterns of Gfap-Calm2. a-c Spatial correlations of Gfap-Calm2 in Allen Brain Atlas (a), raw ST mouse brain (b), and imputed ST mouse brain (c). **d-e** Spatial heatmaps of gene Gfap (d) and Calm2 (e) without imputation. **f-g** Spatial heatmaps of gene Gfap (f) and Calm2 (g) with imputation. **h-i**: Co-expression patterns of gene pair Gfap-Calm2 in (h) pseudo-bulk and (i) single-cell. Correlation and p-value are derived from Spearman's rank correlation test. Source data for pseudo-bulk expression values are provided with the paper in the Source Data file.

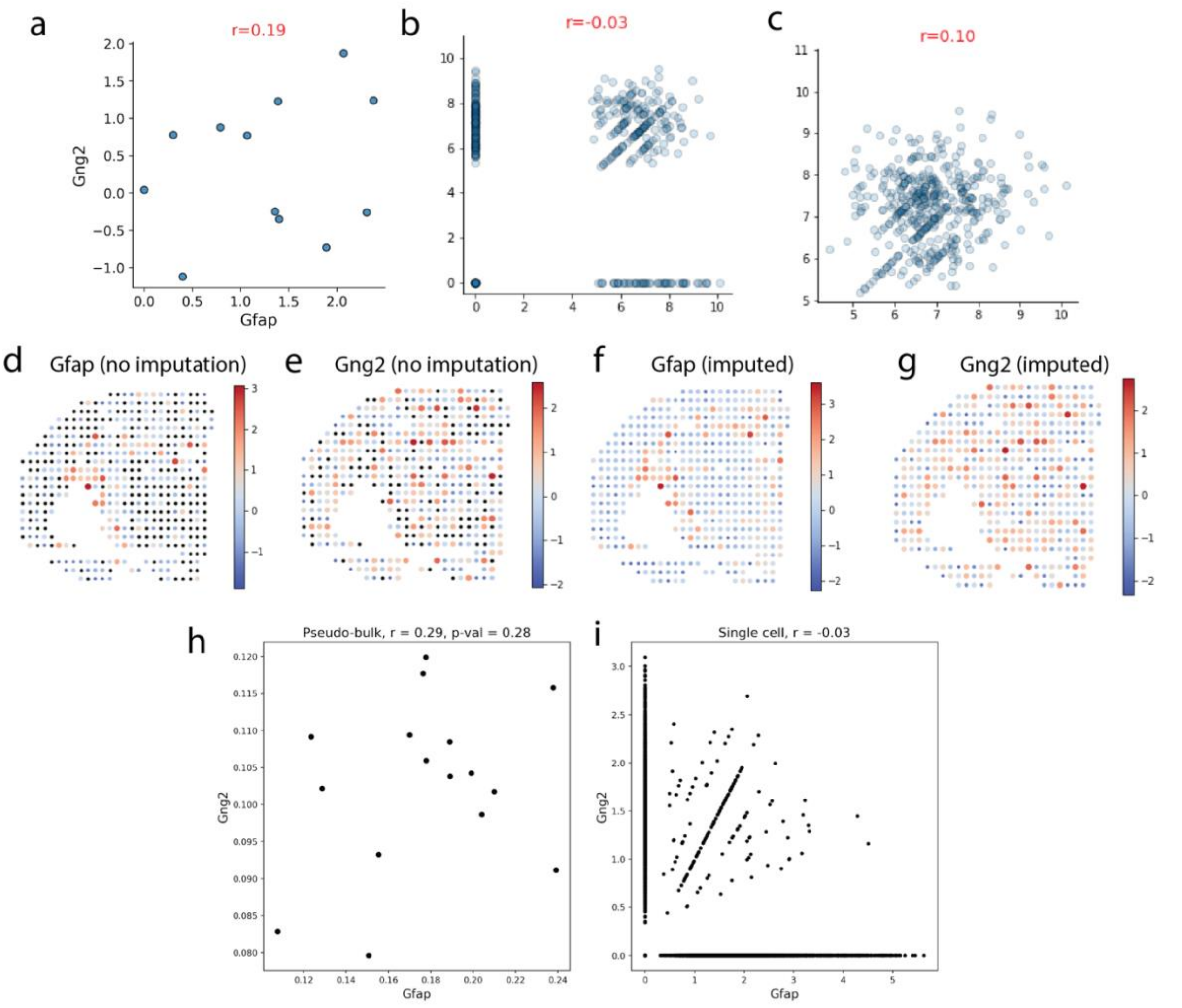

Supplementary Figure 25. Uncorrelated patterns of Gfap-Gng2. a-c Spatial correlations of Gfap-Gng2 in Allen Brain Atlas (a), raw ST mouse brain (b), and imputed ST mouse brain (c). **d-e** Spatial heatmaps of gene Gfap (d) and Gng2 (e) without imputation. **f-g** Spatial heatmaps of gene Gfap (f) and Gng2 (g) with imputation. **h-i** Co-expression patterns of gene pair Gfap-Gng2 in (h) pseudo-bulk and (i) single-cell. Correlation and p-value are derived from Spearman's rank correlation test. Source data for pseudo-bulk expression values are provided with the paper in the Source Data file.

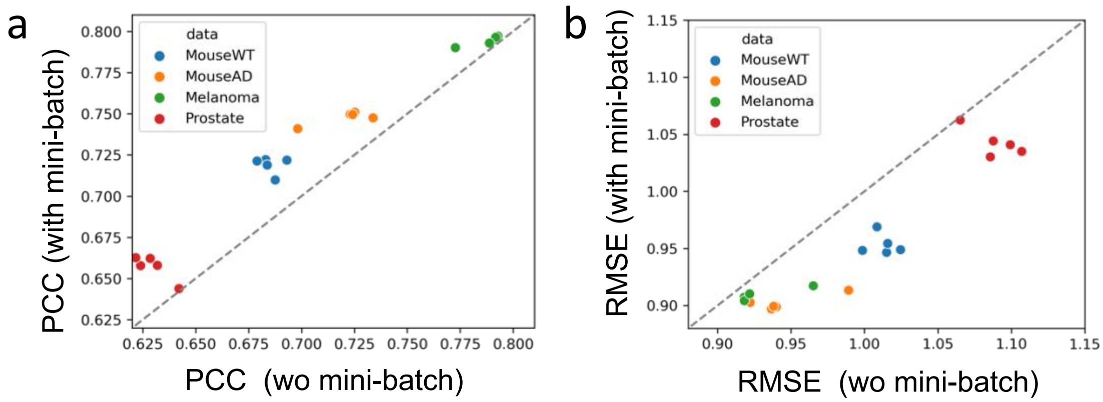

Supplementary Figure 26. Mini-batch improved MIST's imputation performance. Scatter plots of MIST's imputation accuracies comparing without mini-batch (x-axis) and with mini-batch (y-axis) approaches in terms of (a) PCC, Pearson Correlation Coefficient and (b) RMSE, Rooted Mean Square Error. Every point represents a hold-out experiment colored by samples. Source data are provided with the paper in the Source Data file.

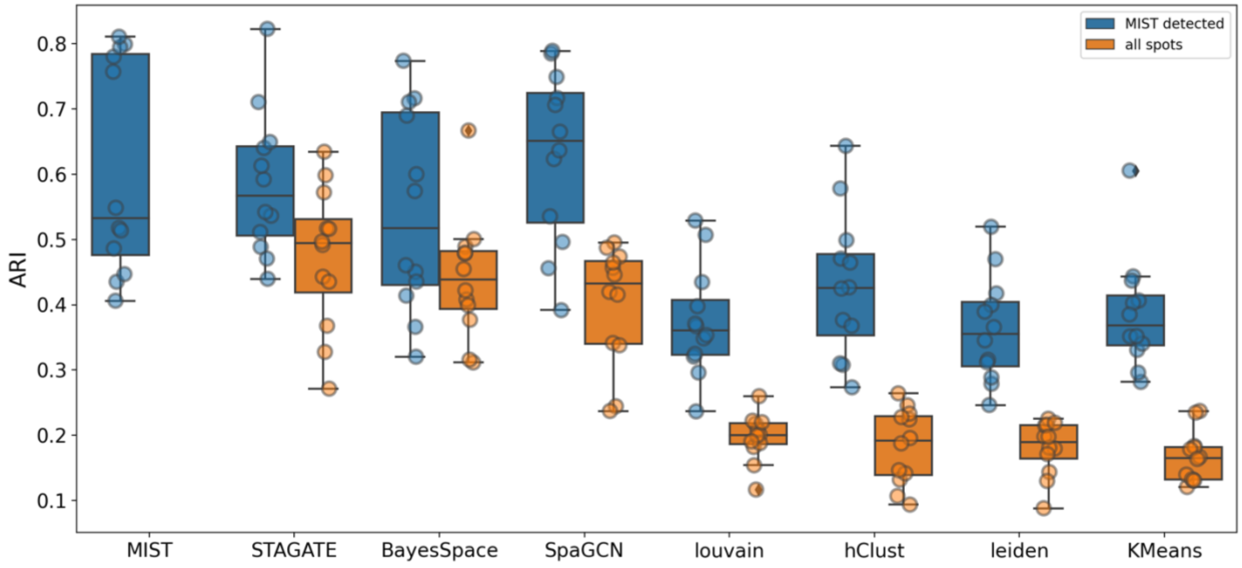

Supplementary Figure 27. Box plots of adjusted rand index (ARI) using different region detection (clustering) methods on 12 independent Human Dorsolateral Prefrontal Cortex samples. ARI scores for MIST were evaluated on MIST detected spots (blue), while other methods were evaluated on both MIST-detected spots (blue) and all spots (orange). Boxplots are defined with center line (median), box limits (upper and lower quartiles) and whiskers that extend at most 1.5 times of the interquartile range. Source data are provided with the paper in the Source Data file.

Algorithm 1: Detect regions under a fixed threshold using the depth first search algorithm

Input :

$G < V, E >$: Graph with vertex set V consisting of M nodes and edge set E with weight set W

ε: Weight threshold to prune the graph G

Output:

R: set of detected rergions

Initialization:

```
E' ← E[W_E'] ≤ ε /* detect low similarity-edges */
E ← E.remove(E') /* filter out low similarity-edges */
R ← {} /* Initialize regions as an empty set */

Function DFS(E, V, v, r)
    r ← r + v
    V ← V.remove(V)
    for u, <u, v> ∈ E do
        if u ∉ r then
            r ← DFS(E, u, r)
        end
    end
return r

while V not empty do
    v ← V.pop()
    r ← DFS(V, E, v, {})
    R ← R + r
end
```

Algorithm 2: Region detection with parameter optimization

Data:

Y: Observed spatial gene expression matrix with M spots and N genes

V: The spot set with M elements

E: The edge set connecting spots $< u, v >$ if spot u and spot v are adjacent

Initialization:

```
E <u,v> ← Pearson(u,v) /* Calculate edge weights.            */
G ←< V,E >
ε ← 0 ;                          /* Set initial threshold.  */
R ← detectRegions(G, ε) /* extract regions under ε using
    Algorithm 1                                               */
t ← getScore(R) /* calculate detection score using eq.(1)   */
for ϵ in [0, 1); stepsize=0.05 do
    R' ← detectRegions(G, ϵ)
    t' ← getScore(R')

    if t' ≤ t then
        t ← t'
        ε ← ϵ
        R ← R'
    end
end
```

Result: R - the final set of detected regions

Algorithm 3: Imputation using region-based mini-batch matrix completion

Input :
Y: Observed spatial gene expression matrix with M spots and N genes
S: set of M spots in the tissue
$\tilde{G}$: set of N genes measured
$R = r_1, r_2, ..., r_n$ `/* n set of MIST-detected regions */`

Output:
X: Imputed spatial gene expression matrix

Initialization:
$S' \leftarrow \bigcup_{\forall r \in R} r$ `/* get all core-region spots */`
$I \leftarrow S - S'$ `/* get all isolated spots */`
$I' \leftarrow randomSplit(I, k)$ `/* randomly split I into k subsets */`
$X' \leftarrow \{\}$

for $r \in R$ **do**
  $\tilde{S} \leftarrow \bigcup_{\forall r_j \neq r} r_j$ `/* combine all spots belong to other core regions */`
  $\tilde{S} \leftarrow randomShuffle(\tilde{S})$ `/* randomly shuffle the set of other core spots */`
  $\tilde{S}' \leftarrow randomSplit(\tilde{R}, k)$ `/* randomly split` $\tilde{R}$ `into k subsets */`
  for $i = 1 : k$ **do**
    $S_i \leftarrow r + I'_k + \tilde{R}'_k$
    $X' \leftarrow X' + MatrixCompletion(Y_{S_i})$
  end
end

`/* Aggregation of results from multiple runs of mini-batch matrix completion */`
$\tilde{X}' \leftarrow averageBySpot(X')$
for $s \in S$ **do**
  $g \leftarrow \forall g \in \tilde{G} : Y_{s,g} == 0$
  $X_{s,g} \leftarrow \tilde{X}'_{s,g}$
end

Supplementary Table 1: Data sets used in region detection and hold-out performance evaluation

Data	Abbreviation	Number of spots (after QC)	Number of genes (after QC)	Sparsity (Zero %)	Source or reference
Human Prostate Cancer, Acinar Cell Carcinoma (FFPE)	HPCACCF	2982	15211	59.2	HPCACCF
Human Breast Cancer (Block A Section 1)	HBCB1	3722	19683	71.0	HBC1
Human Breast Cancer (Block A Section 2)	HBCB2	3907	19693	71.9	HBC2
Human Breast Cancer: Ductal Carcinoma In Situ, Invasive Carcinoma (FFPE)	HBCDC	2467	14960	67.6	HBCDC
Human_Prostate_Cancer_Adenocarcinoma_FFPE	HPCA	4283	15067	68.2	HPCA
Invasive Ductal Carcinoma Stained With Fluorescent CD3 Antibody	HIDC	4632	17843	83.1	HIDC
Human Ovarian Cancer: Whole Transcriptome Analysis. Stains: DAPI, Anti-PanCK, Anti-CD45	HOC	3414	17998	80.2	HOC
Human Glioblastoma:	HG	3397	19294	77.4	HG

Whole Transcriptome Analysis					
Human Colorectal Cancer: Whole Transcriptome Analysis	HCC	3075	17245	79.1	HCC
MouseWT	MouseWT	447	11238	61.9	GEO: GSE152506
MouseAD	MouseAD	488	12304	58.0	GEO: GSE152506
Melanoma	Melanoma	293	16148	84.9	Thrane, 2018[1]
Prostate	Prostate	406	17678	83.6	Berglund, 2018[3]

Supplementary Table 2: region detection performance summary table (Transcriptome) . Statistical significances were derived from two-side t-test without multiple-comparison adjustments. Significance is indicated by *.

Model	Median TSC	ΔTSC (median)	ΔTSC %	P-value (TSC)
MIST	**0.26**	-	-	-
SpaGCN	0.08	-0.15	56	0.02*
STAGATE	0.04	-0.17	66	7×10^{-4}*
BayesSpace (C)	0.06	-0.19	70	8×10^{-4}*
BayesSpace (I)	0.07	-0.16	62	0.005*
HClust	0.12	-0.07	27	0.08
Louvain	0.12	-0.09	33	0.047*
Leiden	0.10	-0.11	42	0.02*
KMeans	0.19	-0.07	25	0.43

Supplementary Table 3: region detection performance summary table (Spatial) . Statistical significances were derived from two-side t-test without multiple-comparison adjustments. Significance is indicated by *.

Model	Median SSC	ΔSSC (median)	ΔSSC %	P-value (SSC)
MIST	**0.26**	-	-	-
SpaGCN	0.09	-0.15	57	0.06
STAGATE	0.04	-0.19	74	0.01
BayesSpace (C)	0.06	-0.13	51	0.06
BayesSpace (I)	0.08	-0.18	71	0.04*
HClust	-0.03	-0.24	94	0.005*
Louvain	-0.05	-0.26	101	0.0004*
Leiden	-0.08	-0.25	98	0.0003*
KMeans	-0.03	-0.28	109	0.006*

Supplementary Table 4: region detection performance summary table (Combined) . Statistical significances were derived from two-side t-test without multiple-comparison adjustments. Significance is indicated by *.

Model	Median CSC	ΔCSC (median)	ΔCSC %	P-value (CSC)
MIST	**0.48**	-	-	-
SpaGCN	0.23	-0.26	54	0.007*
STAGATE	0.06	-0.35	73	0.0002*
BayesSpace (C)	0.08	-0.40	83	0.002*
BayesSpace (I)	0.13	-0.32	68	0.004*
HClust	0.09	-0.22	46	0.004*
Louvain	0.10	-0.33	69	0.0002*
Leiden	0.01	-0.39	81	0.0001*
KMeans	0.15	-0.15	31	0.01*

Supplementary Table 5: Statistical comparing region detection methods' ARIs on Human Dorsolateral Prefrontal Cortex data sets. Statistical significances were derived from two-side t-test without multiple-comparison adjustments. Significance is indicated by *.

Compared methods	Used Spots	P-value	Average ΔARI
STAGATE	MIST spots	0.68	0.02
BayesSpace	MIST spots	0.20	0.07
SpaGCN	MIST spots	0.71	-0.02
hClust	MIST spots	0.0001*	0.18
KMeans	MIST spots	0.0002*	0.22
leiden	MIST spots	0.0003*	0.25
louvain	MIST spots	5.82E-05*	0.23
STAGATE	All spots	0.06	0.14
BayesSpace	All spots	0.01*	0.17
SpaGCN	All spots	0.003*	0.21
hClust	All spots	1.00E-06*	0.42
KMeans	All spots	4.57E-07*	0.44
leiden	All spots	3.34E-06*	0.43
louvain	All spots	3.20E-06*	0.41

Supplementary Table 6: Statistical comparing region detection methods' ARIs improvements on MIST-detected spots using Human Dorsolateral Prefrontal Cortex data sets. Statistical significances were derived from two-side t-test without multiple-comparison adjustments. Significance is indicated by *.

Method	ARI (all spots)	ARI (MIST spots)	Average ΔARI	P-value
STAGATE	0.47	0.58	0.112	0.001
BayesSpace	0.44	0.54	0.101	0.009
SpaGCN	0.4	0.63	0.228	0
hClust	0.18	0.43	0.245	0
KMeans	0.17	0.39	0.219	0
leiden	0.18	0.36	0.182	0
louvain	0.2	0.37	0.177	0

Supplementary Note 1: Additional validation of co-expression gene pairs

Data collection and process

Another single-cell RNA-sequencing cohort, which includes 16 mice brains with expression data for 37089 single cells published by Methodios Ximerakis in 2019[4] (GSE129788) was used to further validate the co-expression patterns of the two pairs of genes, Cldn11-Arhgef10 and Gfap-Aqp4.

Single-cell level co-expression analysis

Spearman correlation coefficient was calculated for each gene pair using the Python package Scipy[5]. Each single cell across the 16 samples were treated as a sample.

Pseudo-bulk-level co-expression analysis

To generate the pseudo-bulk data to reduce the dropout effects in single-cell sequencing, each gene's expression value is estimated by averaging across all the single cells within one mouse brain sample. By doing so, 16 expression values were calculated for each gene. Then, Spearman's correlation coefficients were calculated for Cldn11-Arhgef10 and Gfap-Aqp4 to represent the pseudo-bulk-level co-expression patterns.

Results were summarized in Supplementary Figure 23-25.

References

1. Thrane, K., Eriksson, H., Maaskola, J., Hansson, J. & Lundeberg, J. Spatially resolved transcriptomics enables dissection of genetic heterogeneity in stage III cutaneous malignant melanoma. *Cancer Res.* **78**, 5970–5979 (2018).
2. Chen, W.-T. *et al.* Spatial transcriptomics and in situ sequencing to study Alzheimer's disease. *Cell* **182**, 976–991 (2020).
3. Berglund, E. *et al.* Spatial maps of prostate cancer transcriptomes reveal an unexplored landscape of heterogeneity. *Nat. Commun.* **9**, 1–13 (2018).
4. Ximerakis, M. *et al.* Single-cell transcriptomic profiling of the aging mouse brain. *Nat. Neurosci.* **22**, 1696–1708 (2019).
5. Virtanen, P. *et al.* {SciPy} 1.0: Fundamental Algorithms for Scientific Computing in Python. *Nat. Methods* **17**, 261–272 (2020).
