# Supplementary material for: Region-specific denoising identifies spatial co-expression patterns and intra-tissue heterogeneity in spatially resolved transcriptomics data
Source: Nat Commun. 2022 Nov 14;13:6912. doi: 10.1038/s41467-022-34567-0 (PMC9663444; doi:10.1038/s41467-022-34567-0)
Supplement: Supplementary file 2 — Reporting Summary [file 41467_2022_34567_MOESM2_ESM.pdf]

nature portfolio

Corresponding author(s): Zhadong Liu, PhD

Last updated by author(s): Oct 27, 2022

Reporting Summary

Nature Portfolio wishes to improve the reproducibility of the work that we publish. This form provides structure for consistency and transparency in reporting. For further information on Nature Portfolio policies, see our Editorial Policies and the Editorial Policy Checklist.

Statistics

For all statistical analyses, confirm that the following items are present in the figure legend, table legend, main text, or Methods section.

n/a	Confirmed	
☐	☒	The exact sample size (*n*) for each experimental group/condition, given as a discrete number and unit of measurement
☐	☒	A statement on whether measurements were taken from distinct samples or whether the same sample was measured repeatedly
☐	☒	The statistical test(s) used AND whether they are one- or two-sided<br>*Only common tests should be described solely by name; describe more complex techniques in the Methods section.*
☒	☐	A description of all covariates tested
☐	☒	A description of any assumptions or corrections, such as tests of normality and adjustment for multiple comparisons
☐	☒	A full description of the statistical parameters including central tendency (e.g. means) or other basic estimates (e.g. regression coefficient) AND variation (e.g. standard deviation) or associated estimates of uncertainty (e.g. confidence intervals)
☐	☒	For null hypothesis testing, the test statistic (e.g. *F*, *t*, *r*) with confidence intervals, effect sizes, degrees of freedom and *P* value noted<br>*Give P values as exact values whenever suitable.*
☒	☐	For Bayesian analysis, information on the choice of priors and Markov chain Monte Carlo settings
☐	☒	For hierarchical and complex designs, identification of the appropriate level for tests and full reporting of outcomes
☐	☒	Estimates of effect sizes (e.g. Cohen's *d*, Pearson's *r*), indicating how they were calculated

Our web collection on statistics for biologists contains articles on many of the points above.

Software and code

Policy information about availability of computer code

Data collection	12 Human DLPFC samples were downloaded using spatialLIBD: v1.6.5. (https://zenodo.org/badge/DOI/10.5281/zenodo.4730634.svg)
Data analysis	Python: v3.9<br>MIST: v1.0<br>BayesSpace: v1.4.1<br>SpaceGCN: v1.2.5<br>scanpy: v1.8.2<br>MAGIC: v3.0.0<br>scikit-learn: v1.0.2<br>knn-smoothing: v2.1<br>deepimpute: https://github.com/lanagarmire/deepimpute (version not available, first release)<br>McImpute_scRNAseq: https://github.com/aanchalMongia/McImpute_scRNAseq (version not available)

For manuscripts utilizing custom algorithms or software that are central to the research but not yet described in published literature, software must be made available to editors and reviewers. We strongly encourage code deposition in a community repository (e.g. GitHub). See the Nature Portfolio guidelines for submitting code & software for further information.

Data

Policy information about availability of data

All manuscripts must include a data availability statement. This statement should provide the following information, where applicable:

- Accession codes, unique identifiers, or web links for publicly available datasets
- A description of any restrictions on data availability
- For clinical datasets or third party data, please ensure that the statement adheres to our policy

The melanoma and prostate datasets were obtained from the link (http://www.spatialtranscriptomicsresearch.org/) provided in the original publications. The mouse brain samples were obtained from the GEO database (accession number: GSE152506, sample N06_D2 and B06_E1, https://www.ncbi.nlm.nih.gov/geo/query/acc.cgi?acc=GSE152506). Other Visium datasets were downloaded from 10x Genomics (https://www.10xgenomics.com/resources/datasets). The Human DLPFC data was downloaded by following the instructions from spatialLIBD (http://spatial.libd.org/spatialLIBD/). Source data are provided with this paper in the Source Data file.

Field-specific reporting

Please select the one below that is the best fit for your research. If you are not sure, read the appropriate sections before making your selection.

☒ Life sciences ☐ Behavioural & social sciences ☐ Ecological, evolutionary & environmental sciences

For a reference copy of the document with all sections, see nature.com/documents/nr-reporting-summary-flat.pdf

Life sciences study design

All studies must disclose on these points even when the disclosure is negative.

Sample size	Size of every sample was predefined by the available spots in each ST sample listed in Supp. Table 1.
Data exclusions	No data was excluded.
Replication	Model's accuracy was demonstrated on 13 ST samples from diverse tissues. Biological findings including differentially expressed genes and co-expression patterns were validated by other literatures.
Randomization	This is not relevant to our study because we are presenting a computational method to facilitate data analysis in the field. This study does not include any life science experiment design, such as randomization.
Blinding	This is not relevant to our study because we are presenting a computational method to facilitate data analysis in the field. This study does not include any life science experiment design, such as blinding.

Reporting for specific materials, systems and methods

We require information from authors about some types of materials, experimental systems and methods used in many studies. Here, indicate whether each material, system or method listed is relevant to your study. If you are not sure if a list item applies to your research, read the appropriate section before selecting a response.

Materials & experimental systems

n/a	Involved in the study	
☒	☐	Antibodies
☒	☐	Eukaryotic cell lines
☒	☐	Palaeontology and archaeology
☒	☐	Animals and other organisms
☒	☐	Human research participants
☒	☐	Clinical data
☒	☐	Dual use research of concern

Methods

n/a	Involved in the study	
☒	☐	ChIP-seq
☒	☐	Flow cytometry
☒	☐	MRI-based neuroimaging
