# Supplementary material for: Region-specific denoising identifies spatial co-expression patterns and intra-tissue heterogeneity in spatially resolved transcriptomics data
Source: Nat Commun. 2022 Nov 14;13:6912. doi: 10.1038/s41467-022-34567-0 (PMC9663444; doi:10.1038/s41467-022-34567-0)
Supplement: Supplementary file 3 — Description of Additional Supplementary Files [file 41467_2022_34567_MOESM3_ESM.pdf]

Title: Supplementary Data 1

Description: DEG and GSEA results for Supp. Fig. 2.2-2.10.
